# SENIOR FRIENDLY PACKAGING AND PRODUCT TESTING: FEEDBACK FROM OLDER CONSUMERS

**DOI:** 10.1093/geroni/igz038.2380

**Published:** 2019-11-08

**Authors:** Lisa A Hollis-Sawyer, Alison O’Neil

**Affiliations:** 1 Northeastern Illinois University, Chicago, Illinois, United States; 2 SeniorSelect, Atlanta, Georgia, United States

## Abstract

By 2050, older adults ages 65 or older will account for 83.7 million people in the U.S. population (An Aging Nation: The Older Population in the United States, 2014). It is imperative that products and technologies are designed to accommodate age-related changes that older adults are likely to experience. Given this population surge of older adults, there is a growing interest in identifying consumer products that are usable for older adults or “senior friendly.” Senior-friendly product testing (e.g., Senior Select®) focuses on the usability of various health and consumer products targeted to people with diminishment of any of the following: hearing, vision, taste, touch, smell, mobility & dexterity and /or mental acuity. A usability evaluation study was conducted in three senior living communities located in the Atlanta area. Twenty-nine participants ranged in age from 66 years old to 102 years old. Participants were shown a snack bar product and then asked to use the product themselves to perform a series of prepared tasks. After interacting with the product, participants were asked to share any comments that they had concerning the product. Issues of color contrast between the main packaging and the pull tab, easy of gripping and tearing the wrapper, the labeling of the nutrition information, and the package labeling (should refer to “adult” snack) were reported. Many respondents suggested that senior-friendly package design relates to their health and well-being. Implications toward a wide range of products for older adults of varying ability levels will be discussed.

